# One-year recurrence of stroke and death in Lebanese survivors of first-ever stroke: Time-to-Event analysis

**DOI:** 10.3389/fneur.2022.973200

**Published:** 2022-11-14

**Authors:** Celina F. Boutros, Walaa Khazaal, Maram Taliani, Najwane Said Sadier, Pascale Salameh, Hassan Hosseini

**Affiliations:** ^1^Institut Mondor de Recherche Biomédicale (IMRB)-INSERM U955, Ecole Doctorale Science de la Vie et de la Santé, Université Paris-Est Créteil, Paris, France; ^2^Faculty of Medical Sciences, Neuroscience Research Center, Lebanese University, Hadath, Lebanon; ^3^College of Health Sciences, Abu Dhabi University, Abu Dhabi, United Arab Emirates; ^4^Institut National de Santé Publique, Epidémiologie Clinique et Toxicologie (INSPECT-LB), Beirut, Lebanon; ^5^Faculty of Pharmacy, Lebanese University, Hadath, Lebanon; ^6^University of Nicosia Medical School, Nicosia, Cyprus; ^7^Hôpital Henri Mondor, AP-HP, Créteil, France

**Keywords:** stroke, recurrence, death, cumulative risk rate, Lebanon, survivors, factors, burden

## Abstract

**Background:**

To date, despite the application of secondary prevention worldwide, first-ever stroke survivors remain at imminent risk of stroke recurrence and death in the short and long term. The present study aimed to assess the cumulative risk rates and identify baseline differences and stroke characteristics of Lebanese survivors.

**Methods:**

A prospective longitudinal study was conducted among survivors ≥18 years old who were followed-up for 15 months through a face-to-face interview. Kaplan–Meier method was used to calculate the cumulative rates of stroke mortality and recurrence. Cox-regression univariate and multivariable analyses were performed to identify the predictors of both outcomes.

**Results:**

Among 150 subjects (mean age 74 ± 12 years; 58.7% men vs. 44.3% women; 95.3% with ischemic stroke vs. 4.3% with intracerebral hemorrhage), high cumulative risk rates of stroke recurrence (25%) and death (21%) were highlighted, especially in the acute phase. Survival rates were lesser in patients with stroke recurrence compared to those without recurrence (Log rank test *p* < 0.001). Older age was the main predictor for both outcomes (*p* < 0.02). Large artery atherosclerosis was predominant in patients with stroke recurrence and death compared to small vessel occlusion (*p* < 0.02). Higher mental component summary scores of quality of life were inversely associated with stroke recurrence (*p* < 0.01). Lebanese survivors exhibited the highest percentages of depression and anxiety; elevated Hospital Anxiety and Depression Scale (HADS) scores were seen in those with stroke recurrence and those who died (≥80% with mean HADS scores ≥8). Lower Mini-Mental State Examination scores at the acute phase increased the risk of both outcomes by 10% (*p* < 0.03). Three out of 13 mortalities (23.1%) were presented with early epileptic seizures (*p* = 0.012). High educational level was the protective factor against stroke recurrence (*p* = 0.019). Administration of intravenous thrombolysis decreased the risk of both outcomes by 10% (*p* > 0.05).

**Conclusion:**

Higher rates of stroke recurrence and death were observed in the first year following a stroke in Lebanon. Various factors were identified as significant determinants. Thus, health care providers and officials in Lebanon can use these findings to implement effective preventive strategies to best address the management of these factors to reduce the stroke burden and improve the short and long-term prognosis of stroke survivors.

## Introduction

Stroke is a cerebrovascular disorder characterized by the sudden onset of symptoms and clinical signs caused by the disruption of blood supply to parts of the brain (1). It is a major health concern and is considered one of the most devastating neurological diseases worldwide (2). In Lebanon, as in several countries, stroke is one of the leading causes of death and morbidity (3, 4). Fifteen million people worldwide suffer from stroke annually, of which five million die, and another five million are left permanently disabled, placing a burden on the family and community (5). Normal life for the majority of stroke survivors is disrupted and they experience major disabilities affecting their physical and psychological wellbeing, resulting in long-term invalidity or death.

Stroke recurrence is highly prevalent in survivors and is one of the main functional outcomes in the short and long-term post-first-ever stroke (6). A recent global systematic review by Lin et al. including 37 studies conducted over the last 10 years, including 1,075,014 stroke patients, has shown an increasing pooled stroke recurrence rate ranging from 7.7% at 3 months, 9.5% at 6 months, 10.4% at 1 year to 39.7% at 12 years after the initial stroke (7). Neurological deficit caused by a recurrent stroke is more severe than the initial stroke, with a high percentage of prolonged disability and death. Hence, secondary prevention after the first stroke is crucial to reduce stroke recurrence and mortality (8).

The cause of stroke recurrence and mortality is multifactorial (8, 9). Various international papers have identified modifiable and non-modifiable risk factors, including age (10, 11), gender (12, 13), vascular events like hypertension (HTN) (14), hyperlipidemia and atherosclerotic cardiovascular diseases (15), diabetes mellitus (DM) (16), atrial fibrillation (AF) (17), previous history of cerebrovascular events and stroke subtypes (18), lifestyle factors like smoking habits (19), alcohol consumption (4), use of contraceptive pills, obesity and physical inactivity (18, 20, 21), and psychological complications post-first stroke (22, 23). It is imperative and necessary to identify the patients who are at high risk of stroke recurrence and mortality and who may benefit from a close and regular assessment and rapid implementation of preventive treatments (24).

Although the Global Burden of the Disease tends to provide regular worldwide data regarding stroke burden (25), there is still uncertainty in stroke estimates in low to middle-income countries without national-based health surveillance systems. In recent years, extensive data were published on stroke recurrence and fatality determinants worldwide but there is a scarcity of related studies from the Middle East and North Africa (MENA) region (26–30). However, the burden of stroke in low and middle-income countries, including Lebanon, is higher than in high-income countries and is still rising (31, 32). Stroke types, risk factors, knowledge, and adherence to medication were addressed in various Lebanese papers (2, 3, 33–35) but there are no research studies yet which investigate the recurrence of stroke and death after first-ever stroke. The purpose of this study was to measure the cumulative risk rates of stroke recurrence and death in a time-to-event survival analysis at 3, 6, and 12 months post-first-ever stroke and to identify their determinants among Lebanese first-ever stroke survivors.

## Methods

We followed The Strengthening the Reporting of Observational studies in Epidemiology (STROBE) guidelines for a proper reporting of this work (36).

### Study design and population

An epidemiological observational multicenter prospective longitudinal study was conducted in five private and five public medical centers within two big governorates of Lebanon: Mount Lebanon and Beirut. The study period lasted 15 months, from February 2018 until May 2019. Approval of the protocol from ethics committees of all participating centers was granted before initiating any study procedure while abiding by the World Medical Association Declaration of Helsinki in 2013 (37).

The participants included first-ever ischemic or hemorrhagic stroke survivors who were admitted to the hospitals between February and May 2018. The inclusion criteria were as follows: (1) age ≥18 years, (2) Lebanese nationality, (3) having experienced first-ever stroke, well-identified by the following codes of the International Classification of Diseases-10 (ICD-10) (I60-I64) (38): cerebrovascular accident, stroke, ischemic stroke, hemorrhagic stroke, intracerebral hemorrhage or embolic/cerebral vascular thrombosis, and (4) a diagnosis confirmed clinically and through brain imaging. The exclusion criteria were the following: (1) admission for a recurrent stroke or transient ischemic accident or (2) a medical history of neurological and cognitive disorders. The participants (or their legal representatives) provided written informed consent to be enrolled in the study.

### Sample size

Expected sample size was calculated using the Epi-Info 7 program estimating 116 participants, depending on the stroke prevalence of 3.9% obtained by Jurjus et al. (39). After accounting for missing data and lost follow-up data, a total of 150 subjects were included in the study.

### Study procedures

Written consent from the eligible participants was gathered through an interview conducted by three well-trained investigators. Afterward, the participants were followed up for data collection at 3-, 6-, and 12-months post-stroke.

Clinical information was collected through a data collection form. It included the following: (1) age, gender, place of residence, marital status, number of kids, age of subject's custodian, level of education of the subject and his/her custodian, employment status, number of household members, number of rooms and type of health insurance, (2) lifestyle (eating habits, smoking, practice of physical activity, alcohol and other substances consumption, social support), (3) health indicators (anthropometric indices, family/medical/surgical history, comorbidities, treatment taken by subjects), (4) the disease and its severity (types/subtypes/location/symptoms, length of hospital stay, severity of disease, degree of disability, evaluation of the quality of life (QoL) and (5) the stroke consequences (neuropsychiatric disorders, cognitive disorders, hyperglycemia, fatigue, post-stroke pain, falls, pressure ulcers, pulmonary and urinary infections, deep vein thrombosis, pulmonary embolism, seizures, recurrence of stroke, and death).

### Definitions

The initial Stroke or “*Jalta Dimaghia*,” the Arabic synonym, is the most familiar and most specific term for the disease in Lebanon. According to the World Health Organization, “it is a clinical syndrome consisting of rapidly developing clinical signs of focal (or global in case of coma) disturbance of cerebral function lasting more than 24 h or leading to death with no apparent cause other than that from a vascular origin” (40). Ischemic stroke was classified using the Trial of Org 10172 in the Acute Stroke Treatment (TOAST) criteria, which is divided into five subtypes: (1) large-artery atherosclerosis (LAA), (2) cardioembolism, (CE) (3) small-vessel occlusion (SVO), (4) stroke of other determined etiology (OE), and (5) stroke of undetermined etiology (UE) (41).

Stroke recurrence was the main outcome, which was defined the same criteria as that of the initial stroke. Both ischemic and hemorrhagic stroke recurrences were recorded. Only recurrences that occurred 21 days after the initial event was considered (12). Mortality was defined as death from any cause within 12 months after the first-ever stroke onset. If a patient died within the year of follow-up, the cause of death was researched in the hospital or primary care medical records.

To determine the initial stroke characteristics, the Questionnaire for Verifying Stroke-Free Status (QVSFS) was used. This questionnaire was used to investigate if the subjects ever had the following stroke symptoms: sudden painless weakness on one side of the body, sudden numbness or a dead feeling on one side of the body, sudden painless loss of vision in one or both eyes, sudden loss of one-half of vision, sudden loss of the ability to understand what people are saying; and sudden loss of the ability to express ideas verbally or in writing (42). Stroke severity was measured by the National Institutes of Health Stroke Scale (NIHSS), which identifies the level of consciousness, vision (demonstrated by horizontal eye movements and visual field), facial palsy, motor function extremities, ataxia, sensations, speech dysarthria, or aphasia, and attention to multiple types of stimuli. The scale is divided into 2 levels: <21: non-severe stroke, ≥21: severe stroke; however, a NIHSS cutoff score ≤5 predicts a favorable outcome among survivors during the follow-up periods (43, 44). [Cronbach's alpha of (*r*) = 0.942]. We utilized the validated Arabic translation of NIHSS (45). Disability and dependence in Activities of Daily Living (ADL) were measured by the modified Rankin Scale (mRS), which is the most commonly used scale, with mild disability (independence) graded 0–2 and moderate to severe disability graded ≥3 (46) [Cronbach's alpha of (*r*) = 0.946]. The QoL was assessed by the short form (SF12), which consisted of 12 items including eight scales: physical functioning (PF), role limitations due to physical problems (RP), bodily pain (BP), general health (GH), vitality (VT), social functioning (SF), role limitations due to emotional problems (RE), and perceived mental health (MH), and was divided into two summary scores [physical (PCS) and mental component summaries (MCS)]. They demonstrated the mental and physical functions and overall health-related QoL. PCS and MCS were computed through the scores 12 questions and ranged from 0 (lowest level of health) to 100 (highest level of health) with a cut-off of 50 for PCS and 42 for MCS. The scoring was calculated using the United States (US) norm-based scoring algorithm in the Statistical Package for the Social Sciences software (SPSS) (47, 48). The Arabic version of the SF-12 was used (49). A recent study by Haddad et al. was conducted for the validation of the Arabic version among Lebanese adults (50). The cognitive function was evaluated by the Mini-Mental State of Examination (MMSE), with a total score of 30 points where the cut-off point was set at 24, and a higher score defines a normal cognitive function (51). It has been classified into three levels: 24–30 = no cognitive impairment; 18–23 = mild cognitive impairment; and 0–17 = severe cognitive impairment (52) [Cronbach's alpha of (*r*) = 0.882]. Previous research has validated the use of the Arabic version of MMSE among the Lebanese population (53). The severity of psychological disorders, such as anxiety and depression, was assessed using the Hospital Anxiety and Depression Scale (HADS), which was divided into two scales of seven elements: a scale for depression and a scale for anxiety. Scores ranged from 0 to 7 = normal, 8 to 10 = borderline, 11 to 21 = abnormal (54), [Cronbach's alpha of (*r*) = 0.906], the Arabic validated version was utilized in this study (55). In addition, other scales and scores were utilized, such as the Social Support Rating Scale (SSRS) (56), the Fatigue Severity Scale (FSS) (57), the Modified Ashworth Scale (MAS)(58), “*Douleur Neuropathique4*” (DN4) questionnaire (59), and the Visual Analog Scale (VAS) (60).

### Data processing and analysis

Continuous variables were presented as means ± Standard Deviation (SD) and categorical variables as numbers and percentages. A Survival (Time-to-Event) Analysis was utilized, and the Kaplan–Meier method was used to obtain the cumulative risk rates of stroke recurrence and any cause of death at 3-, 6-, and 12-month follow-up. Univariate and multivariable cox proportional hazards regressions were analyzed to determine the predictors of stroke recurrence and death depending on the time of the event occurrence. The explanatory variables were first tested individually against the dependent variable for the presence of a significant association. Variables for which no significant association was found were removed from the model. Regression analyses were then performed. In the multivariable logistic regression model, we included variables reported in the literature to be associated with 1-year stroke recurrence and death post-stroke considering them as potential confounders, such as age, gender, educational level, and stroke severity, in addition to the variables that showed a significant association at *p* ≤ 0.05 across any category in the univariate analysis. The logistic regression models were examined for the goodness of fit. Deviance values were calculated to analyze how well the model fitted each case. In all cases, it was concluded that the model fit was adequate, and the experimental removal of outliers did not violate the model. The strength of association was interpreted using the adjusted hazard ratio (AHR) with a 95% confidence interval (CI). Statistical significance was set at *p* ≤ 0.05. All these analyses were carried out using the SPSS software, version 25 (SPSS^TM^ Inc., Chicago, IL USA).

### Ethical considerations

The study protocol was reviewed and approved by the ethics committees and directors of the participating hospitals (NEUR-2018-001, HDF-1152). Ethical clearance was obtained through a formal letter granted in line with the World Medical Association Declaration of Helsinki in 2013 (37). Written consent was obtained from the subjects after explaining all details of the study. Participants were also informed that there will be no risks or direct benefits from their collaboration with this study. The participation was completely voluntary and enrolled subjects retained the right to withdraw at any time throughout the study. In addition, to maintain confidentiality, all data were coded in the questionnaire, and the materials will be discarded once the legal retention period expired.

## Results

### Baseline characteristics

**Figure 1:**
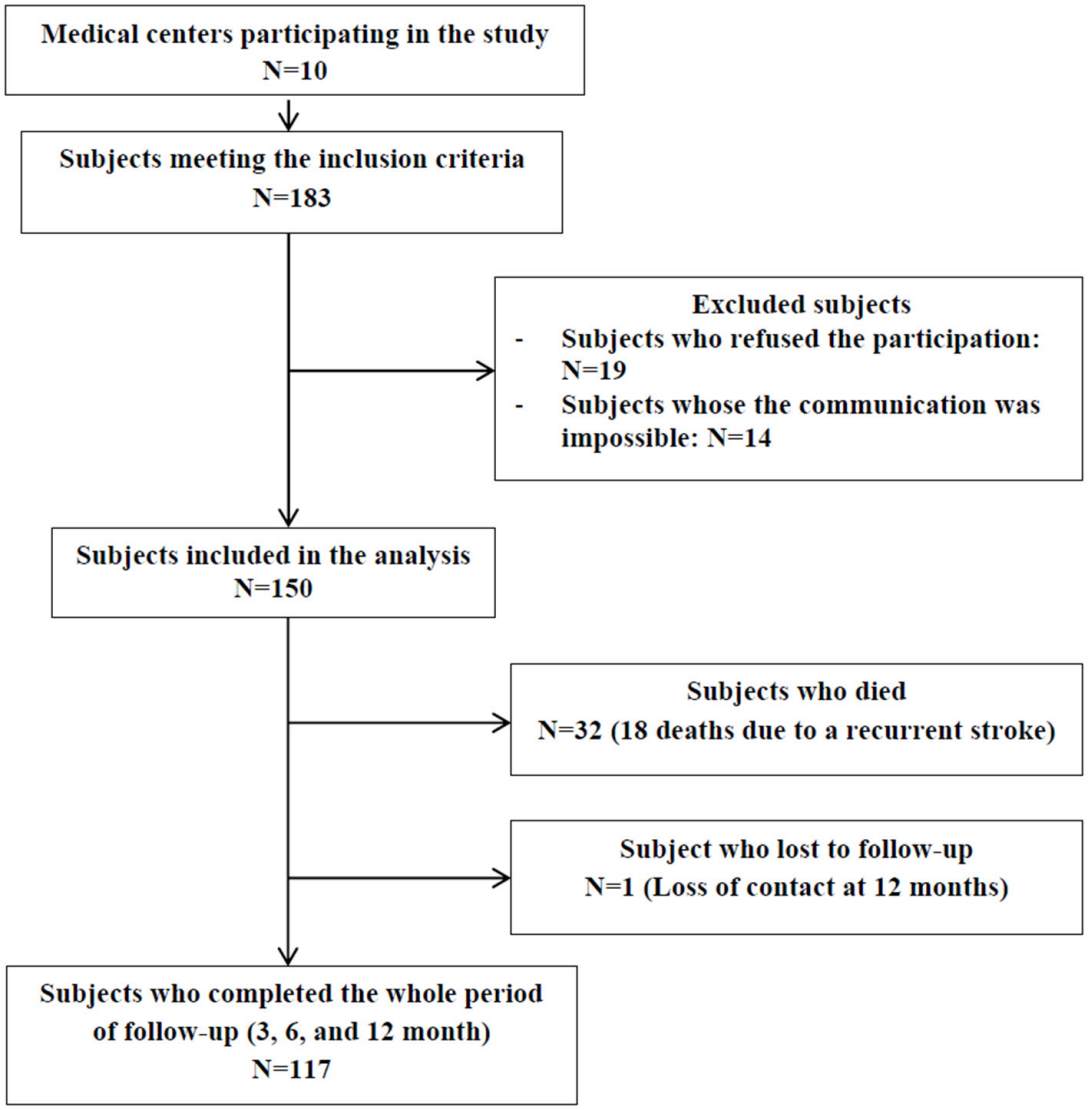
Flow diagram of the steps followed to obtain the sample of the study.

The study population consisted of 150 participants admitted to 10 medical centers in Mount Lebanon and Beirut between February and May 2018. A total of 117 subjects completed the whole follow-up period (3-, 6-, and 12-month), 32 died and one was lost to follow-up at 12 months post-stroke (Figure 1).

Baseline characteristics for all patients, no stroke recurrence/no death groups, and stroke recurrence/death groups are shown in Table 1. The participants had a mean age of 73.69 ± 12.11 years. The population included 88 men (58.7%) and 62 women (44.3%). Stroke recurrence and death were high in subjects with old age (51.4 and 59.4%, respectively), with low educational level (91.4 and 84.4%, respectively), with no employment post-stroke (94.3 and 100%, respectively), and in subjects with a sedentary duration of ≥12 h (58.3 and 83.3%, respectively).

**Table 1:** Baseline characteristics of the study population.

**Baseline characteristics**	**Overall *N* (%) or Mean (±SD)**	**No stroke recurrence *N* (%) or Mean (±SD)**	**Stroke recurrence *N* (%) or Mean (±SD)**	**No death *N* (%) or Mean (±SD)**	**Death *N* (%) or Mean (±SD)**
**Gender**					
Male	88 (58.7)	64 (55.7)	24 (68.6)	71 (60.2)	17 (53.1)
Female	62 (41.3)	51 (44.3)	11 (31.4)	47 (39.8)	15 (46.9)
**Mean age**	73.69 (±12.11)	72.56 (±12.17)	77.42 (±11.28)	72.05 (±11.37)	79.77 (±12.98)
**Age group**					
30–39 years	1 (0.7)	1 (0.9)	0 (0.0)	0 (0.0)	1 (3.1)
40–49 years	7 (4.7)	6 (5.2)	1 (2.9)	7 (5.9)	0 (0.0)
50–59 years	15 (10.0)	11 (9.6)	4 (11.4)	13 (11.0)	2 (6.3)
60–69 years	30 (20.0)	26 (22.6)	4 (11.4)	28 (23.7)	2 (6.3)
70–79 years	43 (28.7)	35 (30.4)	8 (22.9)	35 (29.7)	8 (25.0)
80–89 years	47 (31.3)	31 (27.0)	16 (45.7)	34 (28.8)	13 (40.6)
90–99 years	7 (4.7)	5 (4.3)	2 (5.7)	1 (0.8)	6 (18.8)
**Marital status**					
Single/widowed/divorced	33 (22.0)	29 (25.2)	4 (11.4)	29 (24.6)	4 (12.5)
Married	117 (78.0)	86 (74.8)	31 (88.6)	89 (75.4)	28 (87.5)
**Area**					
Urban	140 (93.3)	106 (92.2)	34 (97.1)	108 (91.5)	32 (99)
Rural	10 (6.7)	9 (7.8)	1 (2.9)	10 (8.5)	0 (0.0)
**Household members**					
Living alone	5 (12.5)	2 (1.9)	3 (12.5)	4 (3.4)	1 (10.0)
Living with family members	123 (96.1)	102 (98.1)	21 (87.5)	114 (96.6)	9 (90.0)
**Presence of a caregiver**					
No	98 (65.3)	79 (68.7)	19 (54.3)	79 (66.9)	19 (59.4)
Yes	52 (34.7)	36 (31.3)	16 (45.7)	39 (33.1)	13 (40.6)
**Educational level**					
Illiterate/primary or complementary education	104 (69.3)	72 (62.6)	32 (91.4)	77 (65.3)	27 (84.4)
Secondary or University education	46 (30.7)	43 (37.4)	3 (8.6)	41 (34.7)	5 (15.6)
**Professional status post-stroke**					
Person without any profession/retired	101 (67.3)	76 (66.1)	25 (71.4)	75 (63.6)	26 (81.3)
Unemployed	30 (20.0)	22 (19.1)	8 (22.9)	24 (20.3)	6 (18.8)
Employed	19 (12.7)	17 (14.8)	2 (5.7)	19 (16.1)	0 (0.0)
**Comorbidities**					
AF	47 (31.3)	37 (32.2)	10 (28.6)	39 (33.1)	8 (25.0)
MI	29 (19.3)	21 (18.3)	8 (22.9)	21 (17.8)	8 (25.0)
HTN	116 (77.3)	90 (78.3)	26 (74.3)	90 (76.3)	26 (81.3)
Other CVD^*^	20 (13.3)	17 (14.8)	3 (8.6)	16 (13.6)	4 (12.5)
DM	60 (40.0)	50 (43.5)	10 (28.6)	50 (42.4)	10 (31.3)
DL	78 (52.0)	61 (53.0)	17 (48.6)	62 (52.5)	16 (50.0)
**Social Security**					
No	25 (16.7)	18 (15.7)	7 (20.0)	19 (16.1)	6 (18.8)
Yes	125 (83.3)	97 (84.3)	28 (80.0)	99 (83.9)	26 (81.3)
**BMI**					
Normal (BMI ≤ 25)	53 (41.1)	40 (38.8)	13 (50.0)	48 (41.0)	5 (41.7)
Overweight (26 ≤ BMI ≤ 30)	46 (35.7)	40 (38.8)	6 (23.1)	41 (35.0)	5 (41.7)
Obesity (31 ≤ BMI ≤ 40)	28 (21.7)	21 (20.4)	7 (26.9)	26 (22.2)	2 (16.7)
Morbid obesity (BMI ≥ 41)	2 (1.6)	2 (1.6)	0 (0.0)	2 (1.7)	0 (0.0)
**Mediterranean diet**					
No	20 (16.0)	16 (15.5)	4 (18.2)	19 (16.1)	1 (14.3)
Yes	105 (84.0)	87 (84.5)	18 (81.8)	99 (83.9)	6 (85.7)
**Smoking status**					
Never smoker	60 (40.5)	45 (39.5)	15 (44.1)	44 (37.3)	16 (53.3)
Former smoker	35 (23.6)	28 (24.6)	7 (20.6)	32 (27.1)	3 (10.0)
Current smoker	53 (35.8)	41 (36.0)	12 (35.3)	42 (35.6)	11 (36.7)
**Physical activity practice**					
No daily practice for ≥30 min	32 (69.6)	24 (64.9)	8 (88.9)	30 (68.2)	2 (99)
Daily practice for ≥ 30 min	14 (30.4)	13 (35.1)	1 (11.1)	14 (31.8)	0 (0.0)
**Sedentary duration**					
1–6 h/day	35 (28.0)	32 (31.7)	3 (12.5)	34 (30.1)	1 (8.3)
7–11 h/day	42 (33.6)	35 (34.7)	7 (29.2)	41 (36.3)	1 (8.3)
≥12 h/day	48 (38.4)	34 (33.7)	14 (58.3)	38 (33.6)	10 (83.3)
**Family history of CVD and neurological diseases**					
No	21 (17.2)	17 (16.8)	4 (19.0)	19 (17.1)	2 (18.2)
Yes	101 (82.8)	84 (83.2)	17 (81.0)	92 (82.9)	9 (81.8)
**Family history of stroke**					
No	46 (52.3)	37 (49.3)	9 (69.2)	41 (51.9)	5 (55.6)
Yes	42 (47.7)	38 (50.7)	4 (30.8)	38 (48.1)	4 (44.4)

*N*, Frequency; %, Percentage; SD, Standard Deviation; AF, Atrial Fibrillation; MI, Myocardial Infarction; HTN, Hypertension; CVD, Cardiovascular Diseases; DM, Diabetes Mellitus; DL, Dyslipidemia; BMI, Body Mass Index.

* Other CVD: coronary artery disease, cardiomyopathy, arrhythmia, chronic heart failure, and thoracic aortic aneurysm.

### Stroke characteristics and their severity

The median interval between the onset of stroke symptoms to admission was 2 h (ranging from 0 to 48 h) for all subjects (mean of 3.43 ± 5.94 h). In addition, the median duration of hospital stay was 7 days (ranging from 2 to 45 days) (mean of 9.69 ± 8.35 days) for all subjects.

**Figure 2:**
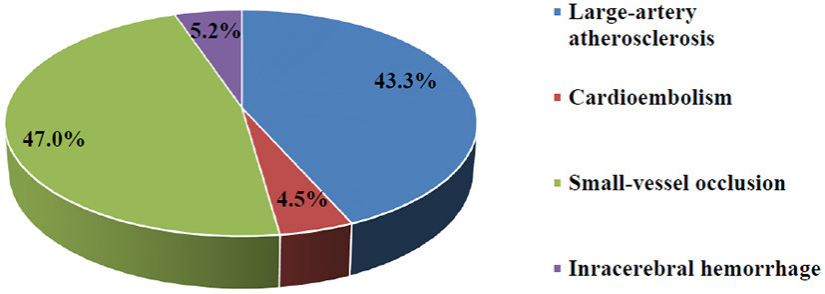
The percentage of stroke types and subtypes according to TOAST classification.

A total of 95.3% of subjects suffered from ischemic stroke compared to 4.7% who suffered from intracerebral hemorrhagic stroke (Figure 2). No subarachnoid hemorrhage was found in the present study. Ischemic stroke cases were categorized into 3 subtypes: LAA (58, 45.7%), CE (6, 4.7%), and SVO (63, 42%). A total of 46.7 and 40% of cases involved the left and right hemispheres, respectively. A majority (70.7%) of subjects were not able to express themselves verbally or in writing at the time of stroke and 70% experienced unilateral weakness.

Stroke severity was estimated as a percentage of NIHSS categories at every follow-up. At 3-month post-stroke, 16.8% of subjects were found to have a severe stroke (NIHSS score ≥ 21). Regarding the degree of disability, a significant proportion of subjects (18%) died (mRS = 6) 3 months post-stroke, whereas, 16% were bedridden (mRS = 5). These percentages decreased from 9.8 and 9.3% (mRS = 5), to 1.6 and 0.8% (mRS = 6) respectively, in the 6- and 12-month follow-ups. The QoL scores are summed up in Table 2, showing decreased PCS and MCS components of QoL (means between 28 and 40) at 3-, 6-, and 12-month follow-up periods. These levels were less than the theoretical averages (cut-off of 50 for PCS and 42 for MCS). At index admission, 47 (31.3%) subjects were already on antiplatelet and anticoagulation agents. At index discharge, these drugs were prescribed to 141 (94%) subjects.

**Table 2:** The quality of life measured by the SF-12 (short form health survey).

**Short form health**	**3 months**	**6 months**	**12 months**
**survey (SF-12)**	**Mean (±SD)**	**Mean (±SD)**	**Mean (±SD)**
**Physical component summary (PCS)**			
General Health (GH)	4.35 (±0.80)	3.78 (±0.97)	3.42 (±1.17)
Physical Functioning (PF)	2.64 (±0.99)	3.22 (±1.29)	3.72 (±1.65)
Role limitations due to physical health (RP)	2.27 (±0.61)	2.50 (±0.81)	2.82 (±0.96)
Bodily Pain (BP)	3.66 (±1.26)	2.94 (±1.24)	2.37 (±1.34)
**PCS**	**28.96 (±7.31)**	**34.92 (±9.21)**	**39.49 (±11.30)**
**Mental component summary (MCS)**			
Vitality (VT)	4.58 (±1.29)	4.15 (±1.37)	3.70 (±1.55)
Social Functioning (SF)	2.22 (±0.89)	2.49 (±1.10)	3.03 (±1.43)
Role limitations due to emotional health (RE)	2.32 (±0.66)	2.50 (±0.81)	2.84 (±0.96)
Mental health (MH)	7.25 (±1.11)	7.10 (±1.23)	7.02 (±1.50)
**MCS**	**32.65 (±9.41)**	**35.17(±10.44)**	**40.12 (±12.85)**

PCS, Physical Component Summary; MCS, Mental Component Summary; SD, Standard Deviation.

### Risk rates of stroke recurrence and death

**Figure 3:**
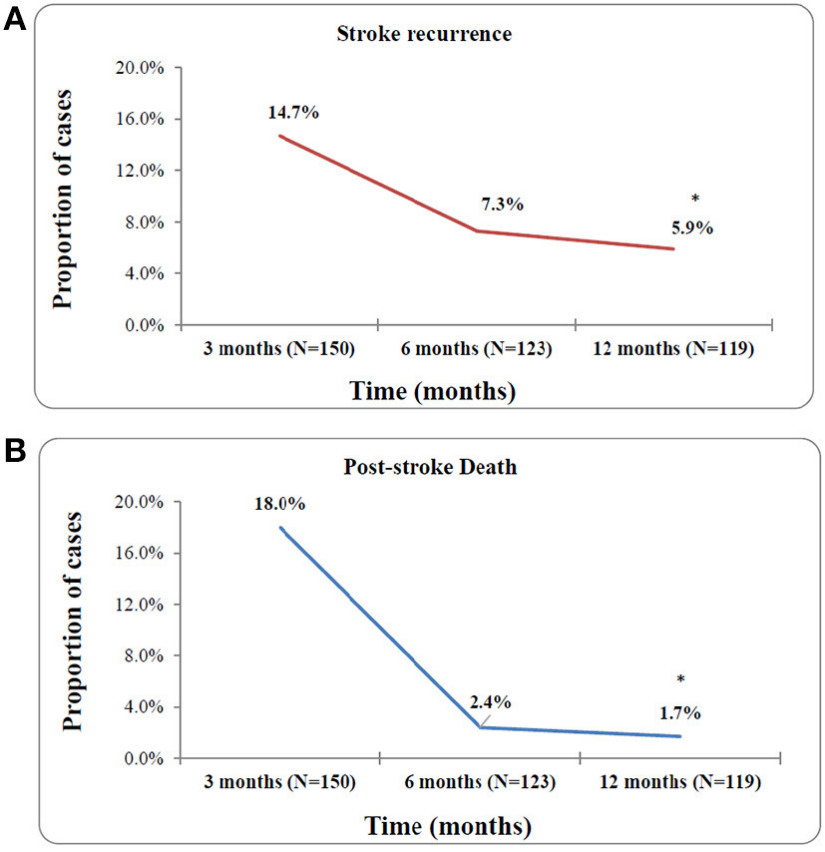
Risk rates of stroke recurrence **(A)** and any-cause of death **(B)** at 3, 6, and 12 month post-stroke. *p-value < 0.001.

Figure 3 shows a high probability of stroke recurrence and death in the first 3 months post-stroke and a significant reduction in these consequences 6 to 12 months (*p* < 0.001) post-stroke. A total of 38 recurrent strokes occurred during the study period, 22 (14.7%) during 3 months post-stroke, nine (7.3%) from 3 to 6 months post-stroke, and seven (5.9%) from 6 to 12 months post-stroke. Additionally, a total of 32 mortalities were reported, 27 (18%) in the 3 months, three (2.4%) in the 3–6 months, and two (1.7%) in the 6–12 months following the first stroke. The reported causes of death were as follows: recurrent stroke (*n* = 18, 56.3%), brain herniation (*n* = 5, 15.6%), myocardial infarction (*n* = 3, 9.4%), complications post-stroke (*n* = 3, 9.4%), ARDS post-stroke (*n* = 1, 3.1%), and pulmonary embolism (*n* = 1, 3.1%).

**Figure 4:**
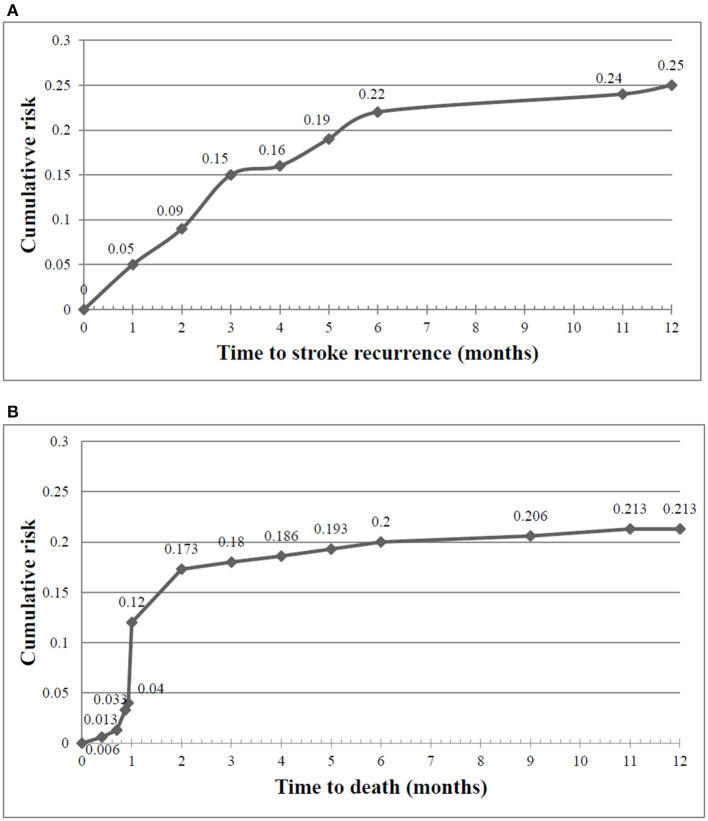
Cumulative risk rates of stroke recurrence **(A)** and any-cause of death **(B)** at 3, 6, and 12 month post-stroke.

**Figure 5:**
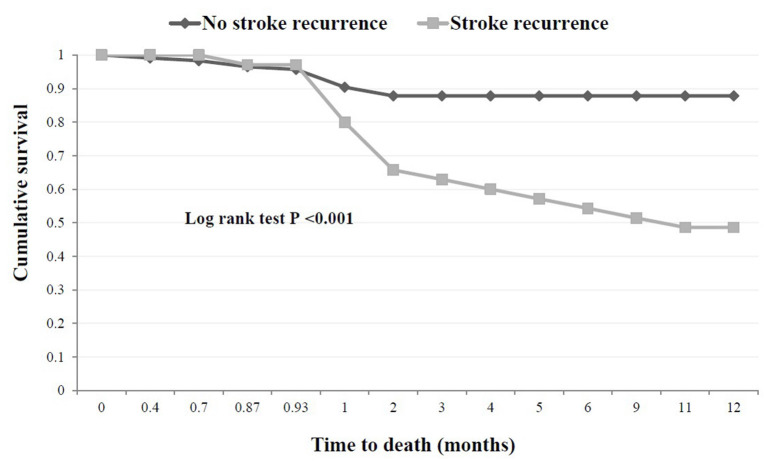
Kaplan Meier estimates of 1-year probability of survival after a first-ever stroke among subjects with and without stroke recurrence. Log rank test *P* < 0.001.

Figure 4 represents the Kaplan–Meier curves of cumulative risk rates over 1 year of follow-up. Cumulative recurrence risk rates among first-ever stroke survivors increased from 15% at the 3 months to 22% at 6 months and 25% at 12 months follow-up. A similar trend was observed for the cumulative any-cause of death risk, which increased from 18% at 3 months to 20% at 6 months to 21% at 12 months post-stroke. The difference between patients with and without 1-year stroke recurrence is shown in Figure 5. The survival rates decreased in patients with stroke recurrence compared to those without recurrence (log rank test *p* < 0.001).

### Predictors of outcomes: Stroke recurrence and any-cause of death

Univariate and Multivariable analyses were performed using Cox proportional unadjusted (UHR) and adjusted hazard ratios (AHR).

#### One-year stroke recurrence predictors

Tables 3, 4 show the UHR of stroke recurrence according to the baseline characteristics, in-hospital course, and post-stroke consequences. Adjusted hazard risks of stroke recurrence are presented in Table 5.

**Table 3:** The association of baseline characteristics with 1-year stroke recurrence using cox proportional hazard regression univariate analysis.

**Baseline characteristics**	**Overall *N* (%) or Mean (±SD)**	**No stroke recurrence *N* (%) or Mean (±SD)**	**Stroke recurrence *N* (%) or Mean (±SD)**	**Unadjusted HR (95%CI)**	***p*-value**
**Age**	73.69 (±12.11)	72.56 (±12.17)	77.42 (±11.28)	1.043 (1.009–1.078)	**0.013**
**Gender, female**	62 (41.3)	51 (44.3)	11 (31.4)	0.698 (0.342–1.424)	0.323
**Marital status, married**	117 (78.0)	86 (74.8)	31 (88.6)	2.417 (0.853–6.848)	0.097
**Secondary or university education**	46 (30.7)	43 (37.4)	3 (8.6)	0.181 (0.055–0.590)	**0.005**
**Stop work after stroke**					
Person without any profession	101 (67.3)	76 (66.1)	25 (71.4)	Ref	
Unemployed	30 (20.0)	22 (19.1)	8 (22.9)	0.929 (0.419–2.061)	0.857
Employed	19 (12.7)	17 (14.8)	2 (5.7)	0.345 (0.082–1.458)	0.148
**Presence of a guardian**	52 (34.7)	36 (31.3)	16 (45.7)	1.6 (0.823–3.113)	0.166
**Living with family members**	123 (96.1)	102 (98.1)	21 (87.5)	0.277 (0.083–0.930)	**0.038**
**Smoking status**					
Non-smoker	60 (40.5)	45 (39.5)	15 (44.1)	Ref	
Ex-smoker	35 (23.6)	28 (24.6)	7 (20.6)	0.630 (0.257–1.546)	0.313
Current smoker	53 (35.8)	41 (36.0)	12 (35.3)	0.801 (0.375–1.712)	0.567
**Daily PA practice for** **≥30 min**	14 (30.4)	13 (35.1)	1 (11.1)	0.259 (0.032–2.074)	0.203
**Sedentary lifestyle**					
1–6 h/day	35 (28.0)	32 (31.7)	3 (12.5)	Ref	
7–11 h/day	42 (33.6)	35 (34.7)	7 (29.2)	1.979 (0.512–7.652)	0.323
≥12 h/day	48 (38.4)	34 (33.7)	14 (58.3)	3.926 (1.128–13.667)	**0.032**
**Moderate level of social support (23** **≤SSRS** **≤44)**	95 (73.1)	79 (74.5)	16 (66.7)	0.768 (0.328–1.794)	0.541
**Mediterranean diet**	105 (84.0)	87 (84.5)	18 (81.8)	0.889 (0.301–2.627)	0.832
**History of AF**	26 (17.3)	21 (18.3)	5 (14.3)	0.832 (0.400–1.733)	0.623
**History of MI**	6 (4.0)	5 (4.3)	1 (2.9)	1.469 (0.667–3.234)	0.340
History of CVD^*^	20 (13.3)	17 (14.8)	3 (8.6)	0.576 (0.176–1.881)	0.361
**History of HTN**	113 (75.3)	88 (76.5)	25 (71.4)	0.855 (0.401–1.826)	0.686
**History of DM**	59 (39.3)	49 (42.6)	10 (28.6)	0.553 (0.266–1.153)	0.114
**History of DL**	74 (49.3)	57 (49.6)	17 (48.6)	0.789 (0.406–1.531)	0.483
**Family history of stroke**	42 (47.7)	38 (50.7)	4 (30.8)	0.470 (0.145–1.527)	0.209

*N*, Frequency; %, Percentage; SD, Standard Deviation; HR, Hazard Ratio; CI, Confidence Interval; Ref, Reference; SSRS, Social Support Rating Scale; AF, Atrial Fibrillation; MI, Myocardial Infarction; HTN, Hypertension; CVD, Cardiovascular Diseases; DM, Diabetes Mellitus; DL, Dyslipidemia.

* CVD, coronary artery disease, cardiomyopathy, arrhythmia, chronic heart failure, and thoracic aortic aneurysm. The bold values indicate significant *p*-values ≤ 0.05.

**Table 4:** The association of stroke in-hospital course and complications post-stroke with 1-year stroke recurrence using Cox Proportional Hazard regression univariate analysis.

**Stroke characteristics**	**Overall *N* (%) or mean (±SD)**	**No stroke recurrence *N* (%) or mean (±SD)**	**Stroke recurrence *N* (%) or mean (±SD)**	**Unadjusted HR (95%CI)**	***p*-value**
**Symptoms during the initial stroke**					
Duration between onset of symptoms and the arrival at the hospital	3.43 (±5.94)	3.62 (±6.39)	2.8 (±4.16)	0.974 (0.908–1.046)	0.474
Sudden painless weakness on one side of the body	105 (70.0)	80 (69.6)	25 (71.4)	1.166 (0.560–2.428)	0.681
Sudden numbness on one side of the body	47 (31.3)	38 (33.0)	9 (25.7)	0.661 (0.310–1.410)	0.284
Sudden painless loss of vision in one or both eyes	23 (15.3)	17 (14.8)	6 (17.1)	1.074 (0.446–2.587)	0.874
Sudden loss of one half of the vision	16 (10.7)	13 (11.3)	3 (8.6)	0.704 (0.216–2.299)	0.561
Sudden loss of the ability to understand what people are saying	74 (49.3)	56 (48.7)	18 (51.4)	1.110 (0.572–2.154)	0.758
Sudden loss of the ability to express verbally or in writing	106 (70.7)	77 (67.0)	29 (82.9)	2.160 (0.897–5.203)	0.086
Administration of Intravenous thrombolysis	9 (6.8)	7 (6.8)	2 (6.9)	0.997 (0.237–4.184)	0.996
Duration of hospital stay	9.69 (±8.35)	8.72 (±7.07)	12.89 (±11.16)	1.041 (1.012–1.070)	**0.004**
ICU stay	63 (42.0)	51 (44.3)	12 (34.3)	0.823 (0.410–1.654)	0.585
**Type of stroke**					
Intracerebral hemorrhage	7 (4.7)	6 (5.2)	1 (2.9)	Ref	
Ischemic stroke	143 (95.3)	109 (94.8)	34 (97.1)	1.468 (0.201–10.727)	0.705
***TOAST classification***					
*LAA*	58 (45.7)	39 (40.2)	19 (63.3)	Ref	
*CE*	6 (4.7)	5 (5.2)	1 (3.3)	0.465 (0.062–3.478)	0.456
*SVO*	63 (49.6)	53 (54.6)	10 (33.3)	0.4 (0.186–0.861)	**0.019**
*OE*	0 (0.0)	0 (0.0)	0 (0.0)		
*UE*	0 (0.0)	0 (0.0)	0 (0.0)		
Right hemisphere	60 (40.0)	44 (38.3)	16 (45.7)	Ref	
Left hemisphere	70 (46.7)	53 (46.1)	17 (48.6)	0.866 (0.437–1.714)	0.679
Bilateral hemisphere	10 (6.7)	8 (7.0)	2 (5.7)	0.708 (0.163–3.080)	0.645
**Treatment post-stroke**					
Use of lipid lowering drug	106 (70.7)	79 (68.7)	27 (77.1)	1.317 (0.598–2.898)	0.494
Use of anti-hypertensive drug	116 (77.3)	90 (78.3)	27 (77.1)	0.983 (0.446–2.164)	0.966
Use of hypoglycemic drug	61 (40.7)	51 (44.3)	10 (28.6)	0.535 (0.257–1.114)	0.095
Use of Antiplatelet agent or Anticoagulant	141 (94.0)	106 (92.2)	35 (99)	22.019 (0.042–11641.17)	0.334
Use of cardiac treatment	66 (44.0)	50 (43.5)	17 (48.6)	1.302 (0.671–2.526)	0.436
**NIHSS at 3 months**					
<21	104 (83.2)	87 (85.3)	17 (73.9)	Ref	
≥21	21 (16.8)	15 (14.7)	6 (26.1)	1.732 (0.683–4.393)	**0.248**
**NIHSS at 6 months**					
<21	109 (73.7)	95 (94.1)	14 (73.7)	Ref	
≥21	11 (9.2)	6 (5.9)	5 (26.3)	3.777 (1.359–10.498)	**0.0.11**
**NIHSS at 12 months**					
<21	109 (94.0)	94 (94.9)	15 (88.2)	Ref	
≥21	7 (6.0)	5 (5.1)	2 (11.8)	2.266 (0.518–9.913)	0.277
**Quality of life at 3 months**					
PCS	28.96 (±7.31)	29.70 (±7.64)	25.68 (±4.35)	0.915 (0.844–0.991)	**0.029**
MCS	32.65 (±9.41)	34.13 (±8.96)	26.09 (±8.67)	0.904 (0.856–0.956)	**<0.001**
**Quality of life at 6 months**					
PCS	34.92 (±9.21)	35.61 (±9.23)	31.22 (±8.35)	0.949 (0.896–1.006)	0.077
MCS	35.17 (±10.44)	36.56 (±10.11)	27.79 (±9.22)	0.920 (0.874–0.969)	**0.002**
**Quality of life at 12 months**					
PCS	39.49 (±11.30)	40.43 (±11.00)	33.71 (±11.76)	0.949 (0.903–0.997)	**0.039**
MCS	40.12 (±12.85)	41.30 (±12.06)	32.88 (±15.42)	0.953 (0.916–0.993)	**0.021**
**Anxiety score post-stroke**					
HADS-A at 3 months	8.20 (±3.90)	7.82 (±3.95)	9.95 (±3.18)	1.132 (1.012–1.265)	**0.030**
HADS-A at 6 months	7.98 (±4.12)	7.64 (±4.21)	9.79 (±3.65)	1.111 (0.997–1.238)	0.056
HADS-A at 12 months	6.46 (±4.75)	6.17 (±4.62)	8.40 (±5.32)	1.085 (0.980–1.201)	0.117
**Depression score post-stroke**					
HADS-D at 3 months	12.15 (±5.31)	11.35 (±5.12)	15.86 (±4.63)	1.166 (1.066–1.276)	**0.001**
HADS-D at 6 months	11.42 (±5.32)	10.72 (±5.09)	15.11 (±5.10)	1.163 (1.058–1.280)	**0.002**
HADS-D at 12 months	9.57 (±6.78)	8.94 (±6.35)	13.80 (±8.20)	1.103 (1.020–1.193)	**0.014**
**Cognitive impairment post-stroke**					
MMSE at 3 months	16.29 (±7.95)	17.18 (±7.78)	12.23 (±7.62)	0.938 (0.889–0.988)	**0.016**
MMSE at 6 months	20.68 (±8.07)	21.90 (±7.14)	14.16 (±9.70)	0.919 (0.876–0.963)	**<0.001**
MMSE at 12 months	23.26 (±7.93)	24.30 (±6.81)	17.12 (±11.01)	0.925 (0.882–0.969)	**0.001**
**Disability degree post-stroke**					
mRS score at 3 months	3.53 (±1.74)	3.21 (±1.714)	4.57 (±1.399)	2.243 (1.643–3.062)	**<0.001**
mRS score at 6 months	2.50 (±1.52)	2.25 (±1.374)	3.64 (±1.677)	1.688 (1.269–2.245)	**<0.001**
mRS score at 12 months	2.16 (±1.55)	1.96 (±1.414)	3.21 (±1.813)	1.518 (1.155–1.994)	**0.003**
**Fatigue post-stroke**					
FSS at 3 months	5.64 (±1.33)	5.51 (±1.38)	6.27 (±0.82)	1.676 (1.068–2.632)	**0.025**
FSS at 6 months	4.90 (±1.44)	4.73 (±1.45)	5.81 (±1.07)	1.746 (1.175–2.593)	**0.006**
FSS at 12 months	3.64 (±2.01)	3.49 (±1.93)	4.67 (±2.23)	1.337 (1.012–1.765)	**0.041**
**Pain level post-stroke**					
Moderate to severe pain at 3 months	75 (61.5)	58 (57.4)	17 (81.0)	2.754 (0.927–8.186)	0.068
Moderate to severe pain at 6 months	57 (48.3)	46 (45.5)	11 (64.7)	1.990 (0.736–5.380)	0.175
Moderate to severe pain at 12 months	31 (27.2)	24 (24.2)	7 (46.7)	2.445 (0.886–6.742)	0.084
**Neuropathic pain score post-stroke**					
DN4 ≥ 4 at 3 months	31 (25.2)	22 (22.0)	9 (39.1)	2.029 (0.878–4.690)	0.098
DN4 ≥ 4 at 6 months	19 (16.0)	16 (16.0)	3 (15.8)	0.947 (0.276–3.250)	0.931
DN4 ≥ 4 at 12 months	10 (8.7)	8 (8.1)	2 (12.5)	1.552 (0.353–6.828)	0.561
**Headache post-stroke (no/yes)**					
Headache at 3 months	45 (36.9)	35 (35.4)	10 (43.5)	1.329 (0.583–3.030)	0.499
Headache at 6 months	33 (28.0)	28 (28.3)	5 (26.3)	0.884 (0.318–2.454)	0.812
Headache at 12 months	17 (14.7)	14 (14.3)	3 (16.7)	1.116 (0.323–3.853)	0.863
**Limb pain (no/yes)**					
Limb pain at 3 months	40 (32.8)	30 (30.3)	10 (43.5)	1.643 (0.720–3.747)	0.238
Limb pain at 6 months	40 (32.8)	30 (30.3)	10 (43.5)	1.643 (0.720–3.747)	0.238
Limb pain at 12 months	20 (17.2)	15 (15.3)	5 (27.8)	1.874 (0.668–5.257)	0.233
**Spasticity score post-stroke**					
MAS ≥ 3 at 3 months	26 (20.3)	20 (19.4)	6 (24.0)	1.175 (0.469–2.942)	0.731
MAS ≥ 3 at 6 months	21 (17.1)	17 (16.7)	4 (19.0)	1.092 (0.367–3.246)	0.874
MAS ≥ 3 at 12 months	13 (11.3)	12 (12.2)	1 (5.9)	0.487 (0.065–3.675)	0.486
**Joint contractures post-stroke (no/yes)**					
Joint contractures at 3 months	45 (36.9)	33 (33.0)	12 (54.5)	2.171 (0.938–5.026)	0.070
Joint contractures at 6 months	38 (32.2)	27 (27.0)	11 (61.1)	3.556 (1.378–9.179)	**0.009**
Joint contractures at 12 months	20 (17.4)	17 (17.2)	3 (18.8)	1.106 (0.315–3.880)	0.875
**Falls at least one time post-stroke**					
At 3 months	49 (37.7)	34 (32.4)	15 (60.0)	2.701 (1.213–6.014)	**0.015**
At 6 months	22 (18.3)	16 (15.8)	6 (31.6)	2.066 (0.785–5.437)	0.142
At 12 months	9 (7.6)	8 (8.0)	1 (5.6)	0.744 (0.099–5.595)	0.774
**Pressure ulcers post-stroke (level ≥1)**					
Pressure ulcers at 3 months	45 (34.6)	31 (29.5)	14 (56.0)	2.635 (1.196–5.806)	**0.016**
Pressure ulcers at 6 months	33 (27.3)	24 (23.8)	9 (45.0)	2.224 (0.921–5.368)	0.076
Pressure ulcers at 12 months	19 (16.1)	13 (13.0)	6 (33.3)	2.701 (1.013–7.204)	**0.047**
Confirmed pneumonia at 3 months post-stroke	27 (20.8)	18 (17.1)	9 (36.0)	2.543 (1.123–5.758)	**0.025**
Confirmed UTI at 3 months post-stroke	36 (27.5)	28 (26.4)	8 (32.0)	1.188 (0.512–2.753)	0.688
Confirmed UTI at 6 months post-stroke	22 (18.0)	14 (13.9)	8 (38.1)	3.068 (1.270–7.411)	**0.013**
Confirmed UTI at 12 months post-stroke	7 (5.9)	6 (6.0)	1 (5.6)	0.890 (0.118–6.685)	0.909
Epileptic seizures at 3 months post-stroke	8 (6.1)	5 (4.7)	3 (12.0)	2.079 (0.622–6.951)	0.234
Epileptic seizures at 6 months post-stroke	9 (7.4)	7 (6.9)	2 (9.5)	1.312 (0.306–5.635)	0.715
Confirmed DVT at 3 months post-stroke	15 (11.5)	10 (9.4)	5 (20.0)	2.125 (0.797–5.664)	0.132
Confirmed DVT at 6 months post-stroke	5 (4.1)	4 (4.0)	1 (4.8)	1.120 (0.150–8.348)	0.912

*N*, Frequency; %, Percentage; SD, Standard Deviation; HR, Hazard Ratio; CI, Confidence Interval; Ref, Reference; ICU, Intensive Care Unit; TOAST, Trial of ORG 10172 in Acute Stroke Treatment; LAA, Large Artery Atherosclerosis; CE, Cardioembolism; SVO, Small-Vessel Occlusion; OE, Other determined Etiology; UE, Undetermined Etiology; NIHSS, National Institutes of Health Stroke Scale; PCS, Physical Component Summary; MCS, Mental Component Summary; HADS-A/HADS-D, Hospital Anxiety and Depression Scale – Anxiety/Depression; MMSE, Mini-Mental State Examination; mRS, modified Rankin Scale; FSS, Fatigue Severity Scale; DN4, “*Douleur Neuropathique* “4 questionnaire; MAS, Modified Ashworth Scale; UTI, Urinary Tract Infections; DVT, Deep Vein Thrombosis. The bold values indicate significant *p*-values ≤ 0.05.

**Table 5:** Independent predictors of 1-year stroke recurrence using cox proportional hazard regression multivariable analysis.

**Variables**	**AHR (95%CI)**	***p*-value**
Education level, secondary or university education	0.164 (0.036–0.745)	0.019
MCS at 3 months	0.927 (0.876–0.980)	0.008
MCS at 6 months	0.904 (0.843–0.969)	0.004
MMSE at 6 months	0.908 (0.831–0.992)	0.033
Depression at 6 months post-stroke	1.176 (1.060–1.305)	0.002

AHR, Adjusted Hazard Ratio; CI, Confidence Interval; MCS, Mental Component Summary; MMSE, Mini-Mental State Examination. All these multivariable analyses included all variables and confounding factors that had a value of *p* ≤ 0.05 in the univariate analysis. The method of selection of the variables which has been chosen here is the backward stepwise method.

The baseline factors associated positively with stroke recurrence were the older age with a mean of 77 ± 11 years [*p* = 0.016, UHR = 1.042, 95% CI (1.008–1.077)], and the sedentary duration of ≥12 h [*p* = 0.032, UHR = 3.926, 95% CI (1.128–13.667)]. Whereas, living with family members was negatively associated with stroke recurrence [*p* = 0.038, UHR = 0.277, 95% CI (0.083–0.930)], and a high educational level was the independent protective factor against stroke recurrence [*p*= 0.019, AHR = 0.164, 95% CI (0.036–0.745)].

Regarding hospital course, survivors with stroke recurrence had a longer duration of hospital stay than those without stroke recurrence (12.89 ± 11.16 days vs. 8.72 ± 7.07 days, respectively) [*p* = 0.004, UHR = 1.041, 95% CI (1.012–1.070)]. Moreover, subjects with SVO had 60% lower risk of stroke recurrence than those with LAA [*p* = 0.019, UHR = 0.4, 95% CI (0.186–0.861)].

Regarding post-stroke consequences, we studied the severity of the stroke, QoL, and functional, mental, neurological, and cognitive outcomes post-stroke. The stroke recurrence was positively associated with severe stroke (NIHSS ≥ 21) at 6-month post-stroke [*p* = 0.011, UHR = 3.777, 95% CI (1.359–10.498)]. Moreover, higher PCS and MCS scores of QoL at 3, 6, and 12 months post-stroke were inversely related to stroke recurrence; however, after adjusting for age and other explanatory factors, the mental dimensions and higher MCS scores at 3- and 6-month follow-up had strong independent opposite relations with stroke recurrence [*p* = 0.008, AHR = 0.927, 95% CI (0.876–0.980); *p* = 0.004, AHR = 0.904, 95% CI (0.843–0.969), respectively]. Similarly, elevated MMSE scores had a significant adjusted low risk of stroke recurrence [*p* = 0.033, AHR = 0.908, 95% CI (0.831–0.992)]. On the other hand, elevated HADS scores for anxiety and depression had a 1-fold increase of the stroke recurrence risk, especially, depression at 6 months post-stroke presented a significant adjusted higher risk [*p* = 0.002, AHR = 1.176, 95% CI (1.060–1.305)].

Furthermore, concerning the functional outcome and post-stroke complications, the high disability degree at 3-, 6-, and 12-months post-stroke predicted a 1-year stroke recurrence, with the largest risk in the acute phase at 3 months. Higher mRS at 3 months post-stroke increased two times the stroke recurrence risk [*p* < 0.001, UHR = 2.243, 95% CI (1.643–3.062); *p* < 0.001]. The following factors affecting the functional outcome were all found as risk factors for 1-year stroke recurrence: fatigue at 3-, 6-, and 12-month post-stroke [*p* = 0.025, UHR = 1.676, 95% CI (1.068–2.632); *p* = 0.006, UHR = 1.746, 95% CI = (1.175–2.593); *p* = 0.041, UHR = 1.337, 95% CI (1.012–1.765), respectively], joint contractures at 6-month post-stroke [*p* = 0.009, UHR = 3.556, 95% CI (1.378–9.179)], falls at least one time at 3-month post-stroke [*p* = 0.015, UHR = 2.701, 95% CI (1.213–6.014)], pressure ulcers (level ≥ 1) at 3- and 12-month post-stroke [*p* = 0.016, UHR = 2.635, 95% CI (1.196–5.806); *p* = 0.047, UHR = 2.701, 95% CI (1.013–7.204), respectively], confirmed pneumonia at 3-month post-stroke [*p* = 0.025, UHR = 2.543, 95% CI (1.123–5.758)], and confirmed urinary tract infections at 6-month post-stroke [*p* = 0.013, UHR = 3.068, 95% CI (1.270–7.411)].

#### One-year any-cause of death predictors

The univariate analysis is tabulated in Tables 6, 7. Table 8 summarizes the multivariable analysis.

**Table 6:** The association of baseline characteristics with 1-year any-cause of death post-stroke using cox proportional hazard regression univariate analysis.

**Baseline characteristics**	**Overall *N* (%) or Mean (±SD)**	**No death *N* (%) Or Mean (±SD)**	**Death *N* (%) Or Mean (±SD)**	**Unadjusted HR (95%CI)**	***p*-value**
Age	73.69 (±12.11)	72.05 (±11.37)	79.77 (±12.98)	1.021 (1.021–1.098)	**0.002**
Female gender	62 (41.3)	47 (39.8)	15 (46.9)	1.303 (0.651–2.610)	0.455
Marital status, Married	117 (78.0)	89 (75.4)	28 (87.5)	2.077 (0.728–5.921)	0.172
Education level, secondary or university education	46 (30.7)	41 (34.7)	5 (15.6)	0.400 (0.154–1.039)	0.060
**Professional status**					
Person without any profession/retired	101 (67.3)	75 (63.6)	26 (81.3)	Ref	
Unemployed	30 (20.0)	24 (20.3)	6 (18.8)	0.714 (0.294–1.736)	0.458
Social security	125 (83.3)	99 (83.9)	26 (81.3)	0.878 (0.361–2.133)	0.774
Presence of a guardian	52 (34.7)	39 (33.1)	13 (40.6)	1.263 (0.624–2.558)	0.516
**Household members**					
Living alone	5 (3.9)	4 (3.4)	1 (10.0)	Ref	
Living with family members	123 (96.1)	114 (96.6)	9 (90.0)	0.379 (0.048–2.990)	0.357
Mediterranean diet	105 (84.0)	99 (83.9)	6 (85.7)	1.128 (0.136–9.367)	0.912
**Smoking status**					
Non-smoker	60 (40.5)	44 (37.3)	16 (53.3)	Ref	
Ex-smoker	35 (23.6)	32 (27.1)	3 (10.0)	0.306 (0.089–1.050)	0.060
Current smoker	53 (35.8)	42 (35.6)	11 (36.7)	0.822 (0.382–1.772)	0.618
**Sedentary lifestyle**					
1–6 h/day	35 (28.0)	34 (30.1)	1 (8.3)	Ref	
7–11 h/day	42 (33.6)	41 (36.3)	1 (8.3)	0.827 (0.052–13.218)	0.893
≥12 h/day	48 (38.4)	38 (33.6)	10 (83.3)	7.768 (0.994–60.693)	0.051
Moderate level of social support (23 ≤ SSRS ≤ 44)	95 (73.1)	86 (72.9)	9 (75.0)	1.108 (0.300–4.095)	0.877
**Comorbidities**					
History of AF	26 (17.3)	21 (17.8)	5 (15.6)	0.703 (0.316–1.564)	0.388
History of MI	6 (4.0)	4 (3.4)	2 (6.3)	1.562 (0.702–3.477)	0.275
History of DL	74 (49.3)	58 (49.2)	16 (50.0)	0.895 (0.448–1.790)	0.755
History of CVD^*^	20 (13.3)	16 (13.6)	4 (12.5)	0.913 (0.320–2.604)	0.865
History of HTN	113 (75.3)	88 (74.6)	25 (78.1)	1.302 (0.536–3.162)	0.561
History of DM	60 (40.0)	50 (42.4)	10 (31.3)	0.683 (0.323–1.442)	0.317
Family history of CVD	101 (82.8)	92 (82.9)	9 (81.8)	0.961 (0.208–4.449)	0.960
Family history of stroke	42 (47.7)	38 (48.1)	4 (44.4)	0.880 (0.236–3.277)	0.849

*N*, Frequency; %, Percentage; SD, Standard Deviation; HR, Hazard Ratio; CI, Confidence Interval; Ref, Reference; SSRS, Social Support Rating Scale; AF, Atrial Fibrillation; MI, Myocardial Infarction; HTN, Hypertension; CVD, Cardiovascular Diseases; DM, Diabetes Mellitus; DL, Dyslipidemia.

* CVD: coronary artery disease, cardiomyopathy, arrhythmia, chronic heart failure, and thoracic aortic aneurysm. The bold values indicate significant *p*-values ≤ 0.05.

**Table 7:** The association of stroke in-hospital course and complications post-stroke with 1-year any-cause of death post-stroke using cox proportional hazard regression univariate analysis.

**Stroke characteristics**	**Overall *N* (%) or mean (±SD)**	**No death *N* (%) or mean (±SD)**	**Death *N* (%) or mean (±SD)**	**Unadjusted HR (95%CI)**	***p*-value**
**Symptoms during the initial stroke**					
Duration between onset of symptoms and the arrival to the hospital	3.43 (±5.94)	3.76 (±6.64)	2.20 (±1.06)	0.933 (0.833–1.045)	0.231
Sudden painless weakness on one side of the body	105 (70.0)	78 (66.1)	27 (84.4)	2.504 (0.964–6.503)	0.059
Sudden numbness on one side of the body	47 (31.3)	42 (35.6)	5 (15.6)	0.376 (0.145 – 1.016)	0.055
Sudden painless loss of vision in one or both eyes	23 (15.3)	19 (16.1)	4 (12.5)	0.777 (0.272–2.214)	0.636
Sudden loss of one half of the vision	16 (10.7)	14 (11.9)	2 (6.3)	0.519 (0.124–2.170)	0.368
Sudden loss of the ability to understand what people are saying	74 (49.3)	55 (46.6)	19 (59.4)	1.519 (0.750–3.075)	0.246
Sudden loss of the ability to express verbally or in writing	106 (70.7)	81 (68.6)	25 (78.1)	1.536 (0.664–3.551)	0.316
Administration of intravenous thrombolysis	9 (6.8)	7 (6.7)	2 (6.9)	0.993 (0.236– 4.177)	0.993
Duration of hospital stay	9.69 (±8.35)	8.58 (±7.63)	13.78 (±9.67)	1.045 (1.016–1.074)	**0.002**
ICU STAY	63 (42.0)	45 (38.1)	18 (56.3)	1.973 (0.981–3.967)	0.057
**Type of stroke**					
Intracerebral hemorrhage	7 (4.7)	5 (4.2)	2 (6.3)	Ref	
Ischemic stroke	143 (95.3)	113 (95.8)	30 (93.8)	1.648 (0.394–6.901)	0.494
**TOAST classification**					
LAA	58 (45.7)	38 (38.4)	20 (71.4)	Ref	
CE	6 (4.7)	4 (4.0)	2 (7.1)	0.991 (0.231–4.241)	0.990
SVO	63 (49.6)	57 (57.6)	6 (21.4)	0.249 (0.100–0.621)	**0.003**
OE	0 (0.0)	0 (0.0)	0 (0.0)		
UE	0 (0.0)	0 (0.0)	0 (0.0)		
**Location of stroke**					
Right hemisphere	60 (40.0)	46 (39.0)	14 (43.8)	Ref	
Left hemisphere	70 (46.7)	55 (46.6)	15 (46.9)	0.916 (0.442–1.897)	0.812
Bilateral hemisphere	10 (6.7)	8 (6.8)	2 (6.3)	0.864 (0.196–3.804)	0.847
Cerebellum	9 (6.0)	8 (6.8)	1 (3.1)	0.456 (0.060–3.469)	0.448
**Treatment post-stroke**					
Use of lipid lowering drug	106 (70.7)	83 (70.3)	23 (71.9)	1.029 (0.476–2.224)	0.942
Use of antihypertensive drug	116 (77.3)	91 (77.1)	26 (81.3)	1.052 (0.455–2.432)	0.906
Use of hypoglycemic drug	61 (40.7)	51 (43.2)	10 (31.3)	0.662 (0.313–1.399)	0.280
Use of antiplatelet agent or anticoagulant	141 (94.0)	111 (94.1)	30 (93.8)	0.848 (0.203–3.549)	0.821
Use of cardiac treatment	66 (44.0)	49 (41.5)	18 (56.3)	1.755 (0.873–3.530)	0.114
**Stroke severity post-stroke**					
NIHSS at 3 months	10.74 (±8.57)	10.43 (±8.32)	15 (±12.04)	1.052 (0.975–1.135)	0.189
NIHSS at 6 months	7.32 (±7.46)	7.19 (±7.32)	15 (±15.56)	1.107 (0.951–1.290)	0.190
**Quality of life at 3 months post-stroke**					
PCS	28.96 (±7.31)	29.31 (±7.36)	23.02 (±1.61)	0.740 (0.552–0.992)	**0.044**
MCS	32.65 (±9.41)	33.26 (±9.21)	22.40 (±6.70)	0.837 (0.729–0.961)	**0.012**
**Quality of life at 6 months post-stroke**					
PCS	34.92 (±9.21)	35.11 (±9.16)	23.44 (±0.80)	0.646 (0.402–1.037)	0.070
MCS	35.17 (±10.44)	35.30 (±10.43)	27.49 (±11.92)	0.921 (0.786–1.081)	0.314
**Cognitive function post-stroke**					
MMSE at 3 months	16.29 (±7.95)	16.64 (±7.90)	8 (±3.39)	0.866 (0.758–0.989)	**0.034**
MMSE at 6 months	20.67 (±8.07)	20.97 (±7.79)	3 (±4.24)	0.738 (0.528–1.032)	0.076
**Anxiety and depression post-stroke**					
HADS-A at 3 months	8.20 (±3.90)	8.08 (±3.91)	11 (±2.55)	1.219 (0.950–1.564)	0.120
HADS-A at 6 months	7.98 (±4.19)	7.97 (±4.22)	8.50 (±2.12)	1.029 (0.741–1.428)	0.865
HADS-D at 3 months	12.15 (±5.31)	11.91 (±5.19)	18 (±5.20)	1.308 (1.028–1.663)	**0.029**
HADS-D at 6 months	11.42 (±5.32)	11.31 (±5.29)	17.50 (±4.95)	1.304 (0.908–1.873)	0.150
Disability post-stroke (mRS at 3 months)	2.98 (±1.43)	2.92 (±1.42)	4.60 (±0.55)	3.568 (1.067–11.926)	**0.039**
Spasticity at 3 months (MAS ≥3)	26 (20.3)	24 (20.3)	2 (20.0)	0.961 (0.204–4.528)	0.960
Joint contractures at 3 months	45 (36.9)	42 (36.2)	3 (50.0)	1.719 (0.347–8.516)	0.507
Falls at least 1 time at 3 months	49 (37.7)	44 (37.3)	5 (41.7)	1.188 (0.377–3.743)	0.769
Confirmed pneumonia at 3 months post-stroke	27 (20.8)	20 (17.1)	7 (53.8)	4.848 (1.629–14.430)	**0.005**
Confirmed UTI at 3 months post-stroke	36 (27.5)	32 (27.1)	4 (30.8)	1.162 (0.358–3.772)	0.803
Epileptic seizures at 3 months post-stroke	8 (6.1)	5 (4.2)	3 (23.1)	4.769 (1.311–17.345)	**0.018**
Confirmed DVT at 3 months post-stroke	15 (11.5)	12 (10.2)	3 (23.1)	2.475 (0.681–8.999)	0.169
Recurrent stroke at 3 months	22 (14.7)	9 (7.6)	13 (40.6)	4.885 (2.398–9.954)	**<0.001**

*N*, Frequency; %, Percentage; SD, Standard Deviation; HR, Hazard Ratio; CI, Confidence Interval; Ref, Reference; ICU, Intensive Care Unit; TOAST, Trial of ORG 10172 in Acute Stroke Treatment; LAA, Large Artery Atherosclerosis; CE, Cardioembolism; SVO, Small-Vessel Occlusion; OE, Other determined Etiology; UE, Undetermined Etiology; NIHSS, National Institutes of Health Stroke Scale; PCS, Physical Component Summary; MCS, Mental Component Summary; MMSE, Mini-Mental State Examination; HADS-A/HADS-D, Hospital Anxiety and Depression Scale – Anxiety/Depression; Mrs, modified Rankin Scale; MAS, Modified Ashworth Scale; UTI, Urinary Tract Infections; DVT, Deep Vein Thrombosis. The bold values indicate significant *p*-values ≤ 0.05.

**Table 8:** Independent predictors of 1-year any-cause of death using cox proportional hazard regression multivariable analysis.

**Variables**	**AHR (95%CI)**	***p*-value**
Age	1.039 (1.002–1.078)	0.040
Length of hospital stay	1.037 (1.006–1.069)	0.018
Depression at 3 months post-stroke	1.302 (1.027–1.650)	0.029
Recurrent stroke at 3 months post-stroke	3.557 (1.679–7.537)	0.001
MMSE at 3 months	0.866 (0.758–0.989)	0.034
Epileptic seizures at 3 months post-stroke	7.313 (1.538–34.768]	0.012

AHR, Adjusted Hazard Ratio; CI, Confidence Interval; MMSE, Mini-Mental State Examination. All these multivariable analyzes included all variables and confounding factors that had a value of *p* ≤ 0.05 in the univariate analysis. The method of selection of the variables which has been chosen here is the backward stepwise method.

The death rate increased within the first year of stroke with the advanced age (mean of 80 ± 13 years) [*p* = 0.040, AHR = 1.039, 95% CI (1.002–1.078)]. Patients who died within the first year post-stroke had a longer duration of hospital stay at index stroke (mean of 13.78 ± 9.67) [*p* = 0.018, AHR = 1.037, 95% CI (1.006–1.069)], and 71.4% (20/28) were affected by LAA ischemic stroke vs. 21.4% with SVO [*p* = 0.003, UHR = 0.249, 95% CI (0.100–0.621)].

Regarding the post-stroke course, various factors were significant. Higher PCS and MCS of QoL scores were inversely associated with the 1-year mortality post-stroke [*p* = 0.044, UHR = 0.740, 95% CI (0.0552–0.992), *p* = 0.012, UHR = 0.837, 95% CI (0.729–0.961), respectively]. Furthermore, higher MMSE scores at 3 months post-stroke were negatively associated with the 1-year mortality [*p* = 0.034, AHR = 0.866, 95% CI = (0.758–0.989)], whereas elevated scores of mRS for disability and HADS for depression 3-month post-stroke were positively associated with mortality within the first year post-stroke. Death cumulative risk rate had a 3-fold increase among subjects with high disability than those without [*p*= 0.039, UHR = 3.568, 95% CI = (1.067–11.926)]. As for the depression that occurred 3 months post-stroke, a higher HADS score was independently associated with a higher risk of 1-year death post-stroke [*p* = 0.029, AHR = 1.302, 95% CI = (1.027–1.650)].

Subjects with confirmed pneumonia 3-month post-stroke significantly had an increased risk of death 1-year death post-stroke [*p* = 0.005, UHR = 4.848, 95% CI (1.629–14.430)].

Moreover, after adjusting for age and other explanatory factors, the risk of death in the first year following initial stroke was independently associated with epileptic seizures at 3-month post-stroke [*p* = 0.012, AHR = 7.313, 95% CI (1.538–34.768)] and with recurrent stroke at 3- month post-stroke [*p* = 0.001, AHR = 3.557, 95% CI (1.679–7.537)].

## Discussion

The current study is the only hospital-based study in Lebanon that provides data on long-term stroke recurrence and death rates over 1 year of follow-up post-first-ever stroke and to identify the associated risk factors. High rates of stroke recurrence (25%) and death (21.3%) in the first year post-stroke were highlighted in our population. Older age was the main predictor of both these outcomes. Subjects with stroke recurrence and death were more likely to have a poor QoL with low scores of MCS and PCS, moderate to severe disability and motor deficit, and severe cognitive impairment, associated with high levels of anxiety and depression. Early recurrent stroke and epileptic seizures were the main independent predictors of 1-year mortality following a stroke.

The cumulative risk rate of stroke recurrence over 1-year of follow-up was 25%, exceeding the 10–20% rates reported in previous studies in different countries, including Japan (61, 62), China (63), Spain (64, 65), U.S. (66–68), U.K. (12), Turkey (69), and Iran (27). This can be explained first by inappropriate re-education and poor knowledge in patients regarding post-stroke healthy habits for survival and improving overall lifestyle to ensure a better QoL and functional outcome. A recent study by Khalil H. et al., 2020 conducted a community-based survey targeting Lebanese adults aged 50 years and above to assess their stroke-related knowledge and concluded that there is a lack of adequate stroke-related knowledge among Lebanese older people (3). Higher levels of education were a significant predictor of better knowledge (3, 70); Almost 69.3% (104/150) of our study population had a low level of education, of whom 32 had experienced a stroke recurrence (32/35, 91.4%). Second, genetic makeup may be a possible reason for high stroke recurrence. Stroke prevalence in Lebanon may be higher than in other developing countries in the region (34) and population aging in Lebanon is higher than in any other Arab country (71, 72). Third, for the background behind this higher recurrence rate, increased vascular risk factors such as HTN, AF, DM, and DL (32, 73, 74) were remarkable in this study but were not statistically significant. The highest rate of recurrence found in this study was in the early stage, which is relatively comparable with the reported rates by previous literature (27, 75–78). Age and stroke severity at the time of the index stroke are important determinants of stroke recurrence and are associated with early and long term prognosis (27).

Regarding mortality post-stroke, the cumulative risk rate of all-cause of mortality was 21.3% at 1-year of follow-up, which was similar to the results of the study by Abdo et al. in Lebanon in 2019 (79). Various studies worldwide have assessed the long-term post-stroke mortality rate. A cumulative risk rate of death of 40.8% 1-year post-stroke was reported in East Africa (80), with rates of 34.5% in Iran (81), 26.9% in Saudi Arabia (82), 15% in China (76), 28% in Brazil (78), 16% in the US (77), 22% to 29% in the UK (83), and 29.4% in Czech Republic (84). Compared with these rates from different countries, the mortality rate over 1-year post-stroke in Lebanon was less than the rate obtained in East Africa, Iran, Saudi Arabia, Brazil, the U.K., and the Czech Republic, but a little greater than those obtained in China and the U.S. Among Middle Eastern countries, Lebanon represents the lowest 1-year fatality rate following a stroke, which might be because of the difference in patient characteristics or the health-care system.

Most of the 32 deaths observed in the follow-up period were caused by cardiac or neurovascular complications. However, recurrence of stroke was responsible for 56% of these deaths in our study, 41% in the 3 months, 9% during the 3 to 6 months, and 6% at 12 months post first-ever stroke, where the possible reason is older age (72% were ≥80 years old). Similar to other results (79, 85–88), stroke recurrence increases the risk of death four times among stroke survivors.

Several factors are known to influence short- and long-term stroke recurrence and mortality.

Age was found as the main predictor of recurrence and death (64, 89). In our population, we found a significantly higher risk of 1-year stroke recurrence and 1-year mortality with advanced age. Elderly individuals were more likely to have a more severe stroke and increased comorbidities, especially HTN, which was found to be higher in those with stroke recurrence but this difference was not statistically significant and could be attributable to the low sample size.

Men were more exposed to stroke recurrence than women (68.6 vs. 31.4%, respectively); however, this difference was statistically significant neither for stroke recurrence nor for death post-stroke. Several studies from U.S., Europe, and China showed similar outcomes for the sexes (68, 90–92).

Similarly, there was no significant association between comorbidities, such as HTN, DM, AF, and dyslipidemia, and stroke recurrence and death within the first year post-stroke. This may be due to the fact that the majority of the patients were on the lipid-lowering and antithrombotic drugs after the stroke; hence, the non-modifiable risk factors were controlled. Saade et al. in 2021, conducted a study to evaluate the adherence to medication in secondary prevention post-stroke and found that 83% of stroke patients were adherent to their medications (35).

The risk of stroke recurrence in subjects with prolonged sitting hours (≥12 h) was four times higher than in those with shorter sitting hours, thus indicating that physical inactivity increases the risk of stroke relapse (93, 94). Most stroke survivors are engaged in physical inactivity and sedentary behavior, due to many barriers including depression, low motivation, poor to moderate social support, and physical impairment (95). The American Heart Association and the American Stroke Association recommend the following: at least 30 min of moderate-intensity physical exercise (i.e., gait, upper extremity function, balance, muscle strength, motor skills, efficiency in self-care, occupational, and leisure-time activities), sufficient to break a sweat or raise heart rate, one to three times a week (93, 95, 96).

Inversely, a higher educational level was a strong independent protective factor against stroke recurrence. Individuals with a higher level of education were 18% less likely to have a secondary stroke over 1 year after the initial stroke. Previous findings suggested that educational level is an important predictor of long-term prognosis of stroke (97, 98). This group of participants understands and has the knowledge of stroke outcome and recurrence risk factors, as well as secondary preventive habits including practicing physical activity, and adopting a healthy lifestyle after stroke i.e., decreased consumption of alcohol and salt and increased consumption of fruits and vegetables, compliance to medications, and relevant rehabilitation process (97, 99).

Living with family members was found to have a significant negative association with 1-year stroke recurrence, which was consistent with previous studies (98, 100). The important step in the continuum of care for stroke survivors is receiving care from family members while living at home (101, 102).

Interestingly, our study highlighted the significant relationships between ischemic stroke subtypes and the cumulative risk rates of stroke recurrence and death, which showed a predominance of LAA stroke subtype in patients with 1-year stroke recurrence and 1-year all-cause of death compared to SVO. We found that almost 63% of patients with stroke recurrence and 71% of deceased patients were affected by LAA at index stroke. One-year mortality and 1-year stroke recurrence were the lowest for SVO stroke. This major difference was also reported by Kolmos et al. in a newly published systematic review comprising 26 studies conducted between 1997 and 2019 worldwide with similar inclusion criteria (103). Pre-existing conditions, specifically vascular risk factors, including HTN, DL, DM, and AF, in LAA patients with stroke recurrence and death were higher than those with SVO stroke in our study and previous studies (103, 104). A study in Egypt showed that SVO was significantly higher among patients with late recurrence (1 year after stroke or more), while LAA was significantly higher among those with early recurrence (within 1 year) of stroke (6).

Although we did not find any statistical significance in the effectiveness of intravenous thrombolysis as a first line treatment in the reduction of stroke recurrence and mortality as per the stroke index in our study, a higher survival rate and a lesser stroke relapse within 1 year were observed in patients who were treated with intravenous thrombolysis. Only nine LAA patients received intravenous thrombolysis, of whom 7 patients aged between 45 and 78 years survived for 1-year post-stroke and were free of stroke recurrence. This finding shed the light on the efficacy of intravenous thrombolysis on post-stroke prognosis (105, 106). Previous studies suggested that the main barrier against receiving intravenous thrombolysis in Lebanon and other developing countries was delayed in-hospital presentation to recombinant tissue plasminogen activator administration (107, 108). A standardized stroke protocol is lacking in Lebanese hospitals and should be implemented (109).

The patients with stroke recurrence or mortality within 1 year post-stroke had prolonged hospital stay at stroke index (initial stroke occurrence) more than those without stroke recurrence or mortality, which was statistically significant in our study (110). Ween et al., in 2000, studied the impact of early recovery rates after stroke on the functional outcome prediction among stroke survivors and found that the length of hospital stay was significantly prolonged in patients with a poor outcome, thus helping us to estimate the stroke prognosis and guide them for efficient rehabilitation programs (111).

Stroke survivors' health-related QoL is one of the important outcomes of rehabilitation. Stroke has a major impact on the QoL of survivors even among those who have no or minimal post-stroke disability (112). Although the outcomes of most patients with minor symptoms, defined by a low NIHSS score, are favorable, the incidence of permanent stroke-related sequelae, recurrent stroke, or medical complications of stroke is still possible (113). Most stroke survivors perceive their QoL as low compared to their pre-stroke status (114). Several factors such as functional status, ADL, anxiety, depression, neurological and cognitive functions, and environmental and other personal factors have been reported to predict the QoL in stroke survivors, which can worsen the long-term prognosis (115–120). In low resource countries (121, 122), such as Lebanon (108, 123, 124), additional factors like health costs, employment status, and emotional disorders have been reported to influence the stroke survivors' QoL.

The present study findings showed low scores of PCS and MCS components of QoL in all subject; however, the scores were lower in survivors with stroke recurrence and those who died over 1 year of follow-up, especially in the early stage.

The MCS of the QoL (SF, MH, RE, and VT) was found as an independent determinant of stroke recurrence. Hence, percentages of anxiety and depression post-stroke, measured by HADS, were 51.2, 48.3, 36.5%, and 77.2, 74.2, and 56.5% at 3-, 6-, and 12-month post-stroke, respectively. A systematic review conducted by Rafsten et al., in 2018, revealed an overall pooled prevalence of post-stroke anxiety disorders of 29.3% during the first year (125). While the present study has shown a high level of post-stroke anxiety among Lebanese survivors compared to the rate in the aforementioned review.

On the other hand, reviews by Ayerbe et al. and Hacket et al., revealed a cumulative rate of post-stroke depression of 33% (126, 127). Furthermore, a systematic review conducted in the MENA region by Kaadan and Larson, included 34 studies with the lowest rates reported in Saudi Arabia (17%), and Iran (18%), whereas, higher rates are reported in Algeria (56.1%), Jordan (64%), and Morocco (73.2%) (128). Lebanese survivors showed the highest rate of post-stroke depression, which is close to the rate in Moroccan people. This could be due to the general poor QoL following stroke among the Lebanese population, lack of proper care, rehabilitation services, and additional training for healthcare professionals on the symptoms of depression. Another possible explanation could be the use of different methods of assessment (123). There is evidence of a strong relationship between the common psychological disorders post-stroke, anxiety and depression, and the stroke recurrence and death over 1 year following stroke (22, 129–131). Elevated HADS scores for anxiety and depression (80–90% with mean HADS scores ≥8) were seen in subjects with stroke recurrence and death.

Other studies have shown that cognitive impairment after stroke increases the risk of long-term stroke recurrence and shortens long-time survival, especially in the acute phase (90, 132, 133). Almost half (53.7%) of the Lebanese stroke survivors complained of severe cognitive impairment (MMSE ≤ 17) in the early stage post-stroke (3 months post-stroke), 28.3% at 6 months, and 18.8% at 12 months post-stroke. Higher MMSE scores were inversely associated with stroke recurrence and death. Furthermore, after adjusting for age and other explanatory factors, Higher MMSE score found to strong protective factor predictor for both outcomes.

Subjects with stroke recurrence were positively associated with an occurrence of a severe stroke 6 months post-stroke and with moderate to severe disability and high mRS scores (85 and 100% with mean mRS scores >3, respectively), which are consistent with previous study results (80, 134, 135). Subjects with motor deficits, such as fatigue (mean FSS scores > 4), joint contractures (61.1%), falls (60%) and pressure ulcers (33–56%) had a greater risk of stroke recurrence as the risk increased by two to three times in them. The control of motor movement in executing ADL is the main problem after stroke and is one of the factors contributing to a low survival' QoL (136). A Swedish study, conducted in 2014 among 35,000 stroke patients (81% with first-ever stroke), followed up at 3 and 12 months, found a 16% decline among survivors, from a level of independence in ADL to a level of dependence in ADL (137). On the contrary, despite the motor deficits mentioned previously that were mainly in the acute phase, our findings reported a slight improvement of the motor function and level of independence from 3 to 12 months of follow-up. These findings are in agreement with the findings of a review by Wondergem et al. conducted in 2017 that included 28 studies (138), and those of Langhorne et al. conducted in 2011 (139).

Pulmonary infections at 3 months post-stroke were positively associated with stroke recurrence and death over 1 year of follow-up. Almost one-third of subjects with stroke recurrence and half of the subjects who died post-stroke presented with early pulmonary infections. In addition, urinary tract infections at 6 months post-stroke were significantly higher in subjects with stroke recurrence (38.1%). It was found that the majority of these subjects (70–80%) had a severe stroke (NIHSS ≥ 21) and increased disability with high mRS scores (mRS ≥ 3), which were similar to the results of previous studies (110, 140). Stroke may affect the immunological status and level of independence of survivors; thus, severe stroke patients are prone to infections leading to post-stroke readmissions because of recurrent aspiration pneumonia and urinary catheterizations. This condition causes an increase in disability, immobility, and elevated inflammatory markers that contribute to atherogenesis and thrombosis, leading to long-term sequelae, recurrent stroke, and subsequent death (110, 140–145).

Finally, epileptic seizures at 3 months post-stroke (8/131, 6.1%) were reported in one-fifth of subjects who died (3/13, 23.1%) over 1 year of follow-up, including two subjects who died after 4–5 months and one subject at 11 months post-stroke. Stroke recurrence was the primary cause of death among the three subjects. When adjusting for age, stroke severity, and recurrence of stroke, epileptic seizures remained associated with mortality post-stroke. The risk of mortality in the first year post-initial stroke was 7-fold higher in subjects with seizures than those without seizures at 3 months post-stroke. Seizures were linked with severe cognitive impairment and with moderate to severe disability post-stroke (60%). The AHR in the current study is higher than the reported HR in previous studies (146–148). This could be explained by the smaller number of patients with epileptic seizures in this study which could have negatively affected the precision of results. Further large cohort studies are needed to confirm our findings.

### Strengths and limitations

This study has several limitations. First, the small sample size recruited following the previous study considering a low prevalence of stroke in Lebanon of 3.9% according to other countries (39). Second, participating hospitals were limited to the regions of Beirut and Mount Lebanon, even though subjects came from all governorates, they were not representative of the overall population of Lebanon. Third, other recurrence correlates, such as carotid artery sclerosis, imaging findings, and medication adherence may need to be studied to provide more insight into the process of recurrence and death. Therefore, this study could function as a preliminary study for stroke recurrence and death post-stroke and their predictive factors among Lebanese survivors.

However, the prospective multicenter longitudinal study design that was conducted may have decreased recall and selection bias. In addition, we used standardized validated reliable international measuring instruments, and the study was performed by highly qualified and well-trained investigators face-to-face with the subjects, which may have lowered the degree of bias usually resulting from self-completed questionnaires. Furthermore, we used the Arabic-validated version of the measuring instruments, which could have prevented information bias. Nevertheless, future studies with larger sample sizes are required to confirm the current study results.

## Conclusion

Stroke recurrence and death were commonly found in the first year post-stroke, with the largest rates recorded in the acute phase. The risk of stroke recurrence in Lebanon is higher compared to those in western and other eastern countries. A large number of the patients died or had recurrent events due to poor functional, neurological, cognitive, and mental prognosis. Lower cognitive scores, and greater neuropsychological, disability, and severity scales were positively associated with both these outcomes among the Lebanese population. Therefore, the primary public goal is to reduce stroke complications. Implementing effective therapies for secondary prevention is necessary in the acute phase (stroke unit management, thrombolytic, and other reperfusion therapies), as well as rehabilitation and long-term follow-up efforts are needed in order to cope with the burden of stroke in people who have developed or survived a stroke.

## Data availability statement

The datasets presented in this article are not readily available because of ethical and privacy restrictions. Requests to access the datasets should be directed to CB, celinaboutros@gmail.com.

## Ethics statement

The studies involving human participants were reviewed and approved by the Ethics Committees and directors of the participating hospitals (NEUR-2018-001, HDF-1152). Ethical clearance was obtained through a formal letter granted in line with the World Medical Association Declaration of Helsinki in 2013. The patients/participants provided their written informed consent to participate in this study.

## Author contributions

HH, PS, and CB contributed to the conception and design of the study. CB, WK, and MT organized the database. CB performed the statistical analysis and wrote the first draft and the sections of the manuscript. All authors contributed to manuscript revision, read, and approved the submitted version.

## Funding

The authors declare that this study received funding from Association Robert Debré pour la Recherche Médicale (ARDRM). The funder was not involved in the study design, collection, analysis, interpretation of data, the writing of this article or the decision to submit it for publication.

## Conflict of interest

The authors declare that the research was conducted in the absence of any commercial or financial relationships that could be construed as a potential conflict of interest.

## Publisher's note

All claims expressed in this article are solely those of the authors and do not necessarily represent those of their affiliated organizations, or those of the publisher, the editors and the reviewers. Any product that may be evaluated in this article, or claim that may be made by its manufacturer, is not guaranteed or endorsed by the publisher.

## Abbreviations

HTN: Hypertension
DM: Diabetes Mellitus
AF: Atrial Fibrillation
MENA: Middle East and North Africa
STROBE: *Strengthening the Reporting of Observational studies in Epidemiology*
ICD-10: International Classification of Diseases-10
QVSFS: Questionnaire for Verifying Stroke-Free Status
NIHSS: National Institutes of Health Stroke Scale
ADL: Activities of Daily Living
mRS: modified Rankin Scale
Quality of life, QoL, SF12: Short form Health Survey 12
PF: physical functioning
RP: role limitations due to physical problems
BP: bodily pain
GH: general health
VT: vitality
SF: Social Functioning
RE: role limitations due to emotional problems
MH: Mental health
PCS: Physical Component Summary
MCS: Mental Component Summary
US: United States
SPSS: Statistical Package for the Social Sciences software
MMSE: Mini-Mental State Examination
HADS: Hospital Anxiety and Depression Scale
SSRS: Social Support Rating Scale
FSS: Fatigue Severity Scale
MAS: Modified Ashworth Scale
DN4: *Douleur Neuropathique4*
VAS: Visual Analogue Scale
SD: Standard Deviation
AHR: Adjusted Hazard Ratio
CI: confidence interval
DL: Dyslipidemia
MI: Myocardial infarction
CVD: cardiovascular diseases
UHR: Unadjusted Hazard Ratio
UK: United Kingdom.
