# Supplementary material for: One-year recurrence of stroke and death in Lebanese survivors of first-ever stroke: Time-to-Event analysis
Source: Front Neurol. 2022 Nov 14;13:973200. doi: 10.3389/fneur.2022.973200 (PMC9702576; doi:10.3389/fneur.2022.973200)
Supplement: Supplementary file 1 [file Table_1.docx]

**Table 1S. Comparison of the present study findings with previous population-based studies versus hospital-based studies.**

| **The present hospital-based study** | **Previous hospital-based studies** | **Previous population-based studies** |
| --- | --- | --- |
| The cumulative risk rate of stroke recurrence over 1-year of follow-up was 25%, exceeding the 10 to 20% rates reported in previous studies in different countries | 18.6% (Kono Y. et al., Japan, 2011) | 3.2% (Takashima N. et al., Japan, 2020) |
|  | 7.6%, 15.1%, and 15.3% in patients with lacunar infarction of SAI, non-lacunar infarction of SAI and MAIs (Wang G. et al., China, 2019) | 9.5% (Modrego P.J. et al., Spain, 2004) |
|  | 9.4% (Allen N.B. et al., USA, 2009) | 5.67% in 2000 to 3.59% in 2013 (Sozener C.B. et al., USA, 2020) |
|  | 3.7% (Kumral E. et al., Turkey, 2014) | 4.7% (Flach C. et al., UK, 2020) |
|  |  | 5.6% (Salehi M.et al., Iran, 2018) |
| Inappropriate reeducation and poor knowledge of patients of the recommendations for a healthy survivor post stroke | Having a higher education level was associated with a greater degree of knowledge of warning signs (Soto-Camara R. et al., Spain, 2020) | There is a lack of adequate stroke knowledge among Lebanese older people (Khalil HM et al., Lebanon, 2020) |
| Increasing of the vascular risk factors such as HTN, AF, DM, and DL which were remarkable in this study and other previous studies |  | Farah R. et al., Lebanon, 2015 |
|  |  | Mansour Z. et al, Lebanon, 2020 |
| The highest rate of recurrence found in this study was in the early stage, which is relatively comparable with the reported rates by previous literature |  | The majority of recurrent events following FES were reported within the first year after the index stroke, with 7 (1.1%) occurring within the first 30 days and 29 (4.6%) occurring between the first month and the first year (Salehi M. et al., Iran, 2018) |
|  |  | 1,709 strokes followed up for 1 year with 72 recurrences at 30 days, and 113 at 3 months (Lovett J.K. et al., UK, 2004) |
| The cumulative risk rate of all-cause of mortality was 21.3% at 1 year of follow-up | Similar cumulative rate of mortality of 22% at 1-year follow-up in Lebanon (Abdo R. et al., Lebanon, 2019) | 15% in China (Chen Y. et al., China, 2020) |
| Compared with the rates from different countries, high income and middle to low-income countries, the mortality rate over 1 year post stroke in Lebanon seems in the midst, less than the rates obtained in East Africa, Iran, Saudi Arabia, Brazil, UK and Czeck Republic but a bit more than China and US. Among Middle Eastern countries, Lebanon represents the lowest 1-year fatality rate following a stroke | 40.8% in Tanzania (Tessua KK et al., Tanzania, East Africa, 2021) | 28% in Brazil (Cabral NL et al., Brazil, 2015) |
|  | 34.5% in Iran (Novbakht H. et al., Iran, 2020) | 22% to 29% in UK (Gulliford MC et al., UK, 2010) |
|  | 26.9% in Saudi Arabia (AlMekhlafi MA et al., Saud Arabia, 2016) | 29.4% in Czech Republic (Bryndziar T. et al., Czech Republic, 2021) |
|  |  | 16% in United States (Hartmann A. et al., Manhattan, US, 2001) |
| Stroke recurrence is one of the main predictors for one-year mortality, similarly to the findings of previous studies | Abdo R. et al., Lebanon, 2019 |  |
|  | Lekoubou A. et al., Cameroon, 2017 |  |
|  | Wang TA et al., Taiwan, 2021 |  |
| Age was found the main predictor of recurrence and death post stroke | Soriano-Tárraga C. et al., Barcelona, 2018 | Modrego P.J. et al., Spain, 2004 |
| No significant sex differences were observed in stroke recurrence and mortality rates | Di Carlo A. et al., Europe, 2003 | Lambert C. et al, USA, 2020 |
|  | Yu C. et al., China, 2015 | Appelros P. et al., Sweden, 2003 |
| Physical inactivity and prolonged sitting hours increase the risk of stroke recurrence as this and other studies have shown | Hou L. et al., China, 2021 | Butler EN et al., USA, 2014 |
| Educational level was found a strong independent predictor of stroke recurrence | Che B et al., China, 2020 | Pennlert J. et al., Sweden, 2017 |
| Living with family members was a protective factor against stroke recurrence | Kucukyazici B. et al., Canada, 2009 | Pennlert J. et al., Sweden, 2017 |
|  |  | Intamas U. et al., Thailand, 2021 |
| Pre-existing conditions, specifically vascular risk factors, including HTN, DL, DM, AF, in LAA patients with stroke recurrence and death were higher than those with SVO stroke in our study and previous studies | ElNady HM et al., Egypt, 2020 | A systematic review of population-based studies between 1997 and 2019: Kolmos M. et al, 2021 |
|  |  | Saber H. et al., Iran, 2017 |
| A non-statistically significant higher survival rate and lesser stroke relapse within 1 year was observed in patients who were treated with IV thrombolysis |  | Thrombolysis with intravenous alteplase is associated with improved long-term survival and functional status after ischemic stroke (Muruet W. et al., UK, 2018) |
| Patients with stroke recurrence or mortality within 1 year post stroke had prolonged hospital stay at stroke index | Ween JE et al., Rhode Island, 2000 | Abreu P. et al., Portugal, 2020 |
| Lower scores of PCS and MCS of the QoL in survivors with stroke recurrence and who died, similarly to previous studies' findings | Palmcrantz S. et al., Sweden, 2014 |  |
|  | Abubakar SA & Isezuo SA, Nigeria, 2012 |  |
|  | Zhu W. & Jiang Y., China, 2019 |  |
|  | Chen CM et al., Taiwan, 2015 |  |
|  | Gurcay E. et al., Turkey, 2009 |  |
|  | Kim K. et al., Korea, 2014 |  |
| In low resource countries, such as Lebanon, additional factors like health costs, employment status, and emotional disorders have been reported to influence stroke survivors’ QoL | Oni OD et al., Nigeria, 2016 |  |
|  | Khalid W. et al., Pakistan, 2016 |  |
|  | Abdo RR et al., Lebanon, 2018 |  |
|  | Abdo R. et al., Lebanon, 2019 |  |
| There is evidence of the strong relation between the common psychological disorders post stroke, anxiety and depression, and the stroke recurrence and death over 1 year following stroke that we highlighted in our results | Systematic reviews: Bartoli F. et al., 2018 Cai W. et al., 2019 Bartoli F. et al., 2013 Mini-review: Zhang S. et al., 2020 | |
| Subjects with stroke recurrence and death were positively associated with severe stroke at 3 and 6 months post initial stroke and with moderate to severe disability | Tessua KK et al., Tanzania, East Africa, 2021 |  |
|  | Mar J. et al., Spain, 2015 |  |
|  | Zhang J. et al., China, 2019 |  |
| A slight improvement of the motor function and level of independence from 3 to 12 months of follow-up |  | Contradictory finding: 16% decline among survivors, from a level of independence in ADL to a level of dependence in ADL from 3 months to 12 months of follow-up (Ullberg T. et al., Sweden, 2015) |
|  | Similar findings: Systematic review by Wondergem R. et al., 2017 Review by Langhorne P. et al., 2011 | |
| Severe stroke patients are prone to infections leading to post-stroke readmissions because of the recurrent aspiration pneumonia and urinary catheterizations, reflecting more disability, immobility, and elevated inflammatory markers that contribute to atherogenesis and thrombosis, in consequence a long term sequelae, recurrent stroke, and then death, similarly to previous literature | Bjerkreim AT et al., Norway, 2015 | Abreu P. et al., Portugal, 2020 |
|  | Klehmet J. et al., Germany, 2009 |  |
|  | Li SJ et al., China, 2016 |  |
|  | Liu Z. et al., China, 2020 |  |
|  | Review by Kumar S. et al., 2010 Review by Santos Samary C. et al., 2016 | |
| Epileptic seizures were found as independent risk factor for mortality 1-year post stroke, in line with previous papers | Burneo JG et al., Canada, 2010 |  |
|  | Castro-Apolo R. et al., USA, 2018 |  |
|  | Review by Reuck J., 2020 | |
